# “The Cannabinoid Receptors”

**DOI:** 10.3797/scipharm.br-10-02

**Published:** 2010-06-30

**Authors:** Gottfried Reznicek

**Affiliations:** Department of Pharmacognosy, University of Vienna, Austria

As research has progressed, the cannabinoid CB_1_ and CB_2_ receptors have expanded significantly in importance within the neuroscience mainstream. In the presented book leading experts introduce newcomers to the cannabinoid field with chapters covering cannabinoid ligand synthesis and structure activity relationships, the molecular pharmacology of the cannabinoid receptors and the endocannabinoid system, and ultimately, the whole animal pharmacology and therapeutic applications for cannabinoid drugs, what is of particular pharmaceutical interest. Adding to those key topics, the book also examines the current direction of the field with chapters on new putative cannabinoid receptors and challenges for future research. As a part of “*The Receptors*” series (edited by Kim A. Neve), this volume highlights the cannabinoid receptors with the most thorough, focused and essential information available. Comprehensive and cutting-edge, “*The Cannabinoid Receptors”* serves as ideal guidebook for Pharmacologists, molecular biologists and neurobiologists covering this fascinating and vital field in a thorough and focused manner.

